# Validation of a new hemifacial spasm grading questionnaire (HFS score) assessing clinical and quality of life parameters

**DOI:** 10.1007/s00702-021-02343-x

**Published:** 2021-05-08

**Authors:** Bettina Wabbels, Ali Yaqubi

**Affiliations:** 1 Department of Ophthalmology, University of Bonn, Ernst-Abbe-Str. 2, 53127 Bonn, Germany; 2 Ophthalmological Center Lippstadt, Wiedenbrücker Str. 31, 59555 Lippstadt, Germany

**Keywords:** Hemifacial spasm, Botulinum toxin, Quality of life, Questionnaire

## Abstract

Validation of the new hemifacial spasm (HFS) questionnaire “HFS score” that captures both clinical (HFS clinical) and health-related quality of life (HRQOL) parameters (HFS subjective) in one tool and comparison with a global HRQOL questionnaire. Cross sectional, prospective validation study including 143 subjects (62 HFS patients and 81 healthy volunteers) from the University Eye Hospital Bonn. Patients were interviewed with the new HFS score and the SF-12 questionnaire prior to and 3 weeks after Botulinum neurotoxin A (BoNT-A) injection. All HRQOL-related questions (HFS subjective) were answered on a visual analogue scale (VAS) ranging from 0 (no complaints) to 100% (maximum complaints) by the patients themselves. Reproducibility was tested in a study extension with 10 patients by repeat interviews (telephone/personal). The new HFS score questionnaire provided a reliable clinical assessment and demonstrated that BoNT-A therapy significantly reduced frequency and severity of eye and cheek spasms (*p* < 0.001; Wilcoxon test). Relevant aspects of HRQOL of HFS patients were assessed with high accuracy and sensitivity. Significant improvements were achieved after BoNT-A injection in five out of eight HRQOL parameters (*p* ≤ 0.02; Wilcoxon test). Cronbach’s alpha of 0.818 demonstrated good internal consistency. Telephone survey provided comparable results to personal interviews. This new sensitive and specific HFS score seems a reliable instrument to monitor BoNT therapy and customize it to the needs of the individual HFS patient—in clinical studies and daily clinical practice.

**Supplementary Information:**

The online version contains supplementary material available at 10.1007/s00702-021-02343-x.

## Introduction

Hemifacial spasm (HFS) is characterised by involuntary, constantly recurring, clonic, and tonic contractions of the facial muscles innervated by the ipsilateral nerve (Wang and Jankovic 1998; Tan et al., 2002). About 10 people out of 100,000 are affected by the disease (Auger an Whisnant 1990; Nilsen et al. 2004), which is usually caused by a compression of the neurovascular facial nerve (Adler et al. 1992; Tan et al. 1999). In contrast to essential blepharospasm, a condition in which spasms of the eyelid occur predominantly bilaterally, HFS is characterised by a mostly unilateral occurrence of spasms of eye and cheek (Cillino et al. 2010). Although visual function in HFS patients is generally less impaired than in patients with essential blepharospasm, HFS can cause a significant psychosocial burden on patients’ lives. Many patients with HFS suffer severely from the disfiguring facial spasms and avoid contact with other people, which often ends in social isolation and depression (Rosenstengel et al. 2012). If left untreated, the symptoms of this chronic disease last a lifetime. Therapeutic options for HFS include microvascular decompression surgery and botulinum injections (BoNT) (Rosenstengel 2012). To date, botulinum neurotoxin has become the symptomatic treatment of choice for facial hemispasm and several clinical studies have confirmed its high effectiveness and safety in this condition (Defazio et al. 2002; Elston 1992; Jitpimolmard et al. 1998; Jost and Kohl 2001; Laskawi et al. 1994; Yoshimura et al. 1992). In the past, several studies have been conducted on BoNT treatment using different assessment tools to measure treatment success (Elston 1992; Tan et al. 2004, 2005; Yoshimura et al. 1992). However, therapy success was often reported as “duration of effect”, i.e., the time between two injections, rather than using a standardised evaluation scale. Altogether, the lack of a standardised classification of clinical symptoms, the variability of the instruments used, and their limited reproducibility make comparisons between studies difficult (Tan et al. 2004, 2005; Wabbels and Roggenkämper 2012; Weiss et al. 2017). In addition, the psychosocial aspects—an important outcome measure for evaluating HFS and success of BoNT therapy—were often not documented at all or only by means of general questionnaires on health-related quality of life (HRQOL). A validated questionnaire that allows easy assessment and monitoring of HRQOL outcomes in HFS patients and has discriminating power to differentiate HFS patients from healthy subjects is the HFS-7 (Tan et al., 2005). However, a significant drawback of the HFS-7 is that questions are not answered on a visual analog scale but using a categorical clinical scale. Although clinical scales were able to differentiate between BoNT and placebo in two controlled trials enrolling patients with hemifacial spasm (Yoshimura et al.^1992^; Park et al. 1993), it remains at least questionable if this tool would be sensitive enough to detect slight differences between different treatment options. Moreover, the HFS-7 does not capture clinical parameters.

Another validated rating tool is the “Hemifacial Spasm Grading Scale” (HSGS) (Tambasco 2019) which represents an objective, quick and reliable scale to assess localisation, frequency and intensity of hemifacial spasms. However, the HSGS score captures only clinical parameters but no HRQOL parameters, which are an essential outcome measure when evaluating HFS and therapy success.

To date, no comprehensive and sensitive rating tool has been established that captures both clinical and subjective HRQOL parameters of hemifacial spasm (HFS), is easy to implement in clinical practice, and thus enabling standardised assessment of HFS and treatment outcome (Wabbels and Roggenkämper 2012; Tambasco N et al. 2019).

The aim of our study was to validate a new, specific and sensitive questionnaire to assess therapy success of BoNT treatment in HFS patients with regard to clinical parameters as well as subjective HRQOL and to compare these results with a global HRQOL-questionnaire.

## Methods

### Patients, BoNT-therapy, conduct and timing of the questionnaire survey

This cross sectional validation study included patients with HFS who were treated in the Botulinum Toxin Consultation at the University Eye Hospital Bonn, Germany, and received injections of BoNT-A. In addition, subjects who did not suffer from HFS were included as control group. These were recruited at the outpatient clinic of the University Eye Clinic in Bonn among persons accompanying patients. It was ensured that the control group matched the patient group in terms of gender and age. Exclusion criteria for both groups were other diseases restricting quality of life, as well as language problems or neurological deficits impeding completion of the questionnaire. The study was approved by the local ethics committee of the “Rheinische Friedrichs-Wilhelms-Universität Bonn” and all participants provided a signed informed consent form for participation in this study. All HFS patients were interviewed with the newly developed HFS score questionnaire and the global SF-12 questionnaire prior to BoNT-A therapy and 3 weeks after injection, because at that time the maximum effect is expected. At baseline, clinical parameters were documented by the examiner taking into account the patient’s medical history and current clinical picture, while at 3 weeks after injection clinical parameters were documented remotely by the patients themselves under telephone guidance, explaining the clinical symptoms of spasms to the examiner. The completed questionnaires were returned by mail to our clinic. In addition, a post-BoNT examination was performed by an experienced investigator at the patients’ re-presentation at our hospital. The participants of the age-matched control group answered the second part of the HFS questionnaire on HRQOL at baseline and 3 weeks later. In order to verify that the results obtained in a telephone interview were consistent with the assessment of an experienced investigator, a study extension was performed, in which 10 HFS patients underwent another therapy cycle and answered the HFS questionnaire three more times: 2 days prior to the next BoNT-A-injection in a telephone interview, then immediately before BoNT-A-injection in a personal interview at the clinic and finally 3 weeks after the injection in another telephone interview. Adverse effects after BoNT-A-therapy were recorded retrospectively on the basis of patient records.

### HFS score questionnaire and SF-12 questionnaire

The newly developed HFS score questionnaire is based on several modified scales from previous studies and is composed of two parts: the first part, hereafter referred to as “HFS clinical”, evaluates clinical severity of the disease, while the second part, hereafter referred to as “HFS subjective” covers subjective HRQOL parameters. Considerations that led to the development of this questionnaire have already been published in detail (Wabbels and Roggenkämper 2012). HFS clinical is based on the Jancovic Rating Scale (JRS) (Jankovic and Orman 1987) evaluating the frequency and severity of eye spasms and has been extended to also evaluate the frequency and severity of cheek spasms. In our clinical experience, spasms of the eye can often be treated more effectively in HFS patients than spasms of the cheek, which then remain a source of embarrassment for patients. Since these have an impact on patients’ perceptions of disease severity or treatment success, it was our aim to develop a clinical rating for the assessment of cheek spasms and to integrate it into the HFS score. The items for cheek involvement were developed based on our clinical experience with HFS patients (Wabbels and Roggenkämper 2012). HFS clinical (first part of the new HFS score questionnaire) allows to rate both the severity and frequency of spasms of the eye as well as cheek from zero (no spasms) to four points (maximum). Thus, a maximum of eight points can be assigned for eye as well as cheek spasms, so a maximum score of 16 points can be achieved in the HFS clinical. The exact description and classification of the categories can be found in the attached questionnaire (see Supplementary file). The second part of our newly developed questionnaire, “HFS subjective”, is based on the HFS-7 questionnaire (Tan 2005). However, in contrast to the HFS-7, the HFS subjective uses a visual analogue scale (VAS) ranging from 0 (no complaints) to 100 percent (maximum complaints) which is subjectively answered by the patients themselves. In addition, a question on general complaints is included and precedes the three questions on functional and the four questions on psychosocial HRQOL. The HFS score questionnaire is available as Supplementary file; for the survey of the patients, a German version of the questionnaire was used.

The SF‐12 (Short-form-12), a shorter version of the SF‐36, is a well-established questionnaire for generic assessment on HRQOL from the patient’s perspective (Bullinger and Kirchberger, 1998). Twelve questions measure different areas of physical and mental health. For statistical evaluation, the questions are recoded and combined into a physical composite score (PCS) and a mental composite score (MCS) with values from 0 to 100, with higher values reflecting a better health status.

### Data analysis

Results are expressed as median and quartiles. Data were analysed using Microsoft Excel Version 2011 und IBM SPSS Statistics Version 23. For determining statistical significance between two groups, the Student’s *t* test, the Wilcoxon test, and the Chi-squared test were performed, depending on data distribution (*p* < 0.05 considered statistically significant). To allow a comparison of the HFS subjective with the SF-12, also for the HFS subjective a physical composite score (mean score from questions 1 to 4) and a mental composite score (mean score from questions 5 to 8) were generated.

## Results

### Demographic and baseline characteristics

A total of 143 subjects were enrolled in the study, including 62 HFS patients. For two patients 3-week data were not documented, as they could not be reached by telephone. Ten patients were therapy naive and received their first BoNT injection at baseline. The demographic and baseline characteristics are summarised in Table 1. The control group included 81 volunteers with a mean age of 66.04 ± 10.63 years, 34 (42.0%) were men and 47 (58.0%) women. There were no statistically significant differences in age and gender between the patient group and the control group (*p* = 0,107, *t* test for age; *p* = 0,235, Chi-square-test for gender).

**Table 1:** Demographic and baseline characteristics

	HFS patients (*n* = 62)
Age (years)	
Mean ± SD (range)	69.21 ± 12.76 (26–92)
Gender, *n* (%)	
Male	20 (32.3)
Female	42 (67.7)
Therapy duration^a^ (years)	
Mean ± SD (range)	8.8 ± 7.2 (0.4–25)
HFS location, *n* (%)	
Right side	21 (33.9)
Left side	41 (66.1)

^a^Of non-therapy naive patients

Of 58 HFS patients with documented adverse effects, 48 exhibited no adverse effects. Three patients had lagophthalmus, and two additionally suffered from sicca symptoms (burning, tears, and pain). Two patients exhibited a hematoma after injection, one also had epiphora. Four patients showed isolated sicca symptoms. One patient experienced reading difficulty after BoNT therapy.

### Clinical parameters according to HFS clinical

**Fig. 1:**
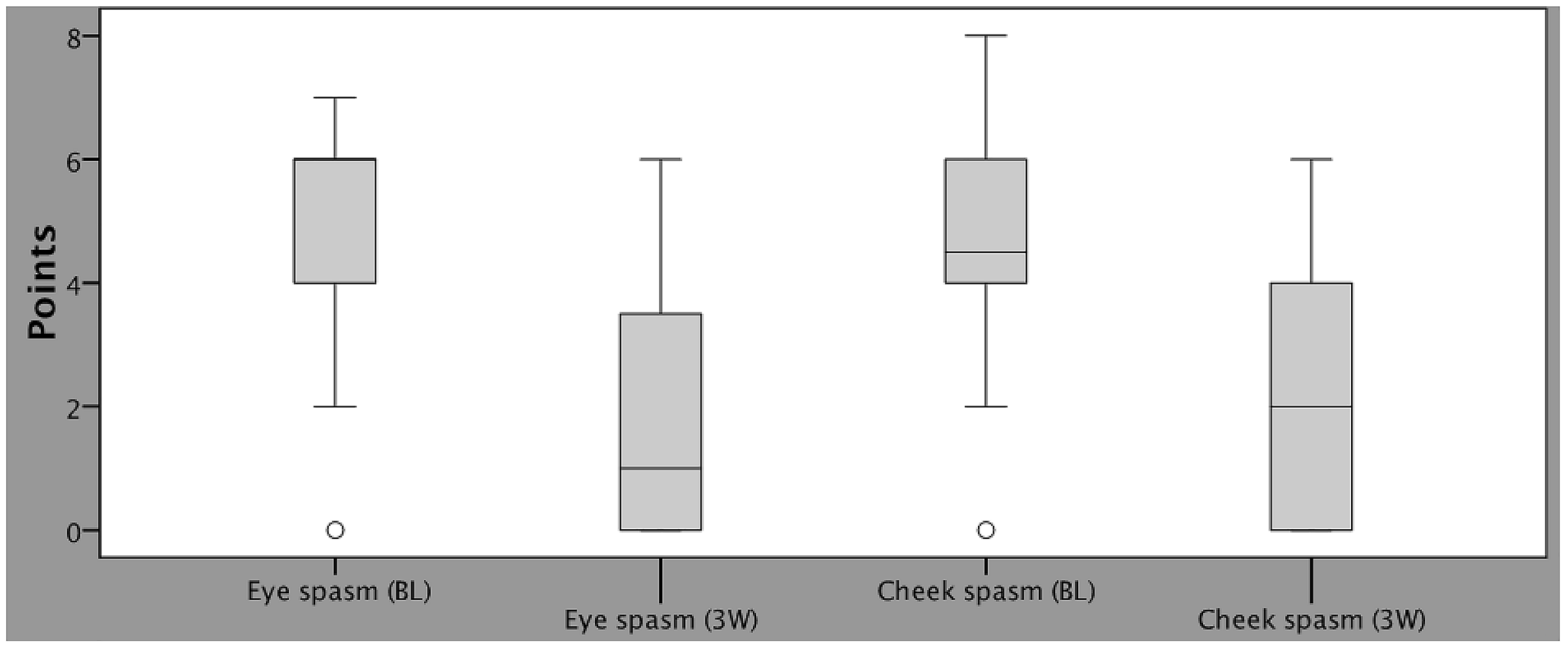
Box plots with whiskers (1.5 × interquartile range (IQR)) of the HFS clinical results, reflecting severity and frequency of spasms of the eye and cheek on a scale from 0 (no spasm) to 8 (maximum expression) points, at baseline (BL) and 3 weeks (3 W) after BoNT-A injection. O indicate mild outliers < 3 × IQR

The assessment of clinical parameters using the HFS clinical (first part of HFS score questionnaire) demonstrated that BoNT therapy significantly reduced frequency and severity of spasms of both the eye and cheek (*p* < 0.001; Wilcoxon test). For spasms of the eye, an improvement from in median 6 points at baseline to 1 point after 3 weeks was observed, while spasms of the cheek improved from in median 5 to 2 points after 3 weeks (scale 0–8) (Fig. 1). The total spasm score (scale 0–16), expressing frequency and severity of both the eye and cheek, improved from in median 10 to 3 points 3 weeks after BoNT-therapy. BoNT–naive and pre-treated patients demonstrated only minor and statistically insignificant differences in clinical parameters at baseline and after BoNT-therapy.

### HRQOL parameters according to HFS subjective

**Fig. 2:**
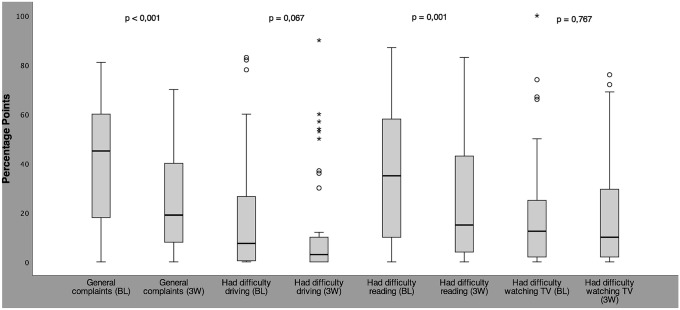
Box plots with whiskers (1.5 × interquartile range (IQR)) of the HFS subjective results on general and functional HRQOL on a scale from 0 (no) to 100 (maximum complaints)% points at baseline (BL) and 3 weeks (3 W) after BoNT-A injection. O indicate mild outliers < 3 × IQR; *indicate extreme outliers

**Fig. 3:**
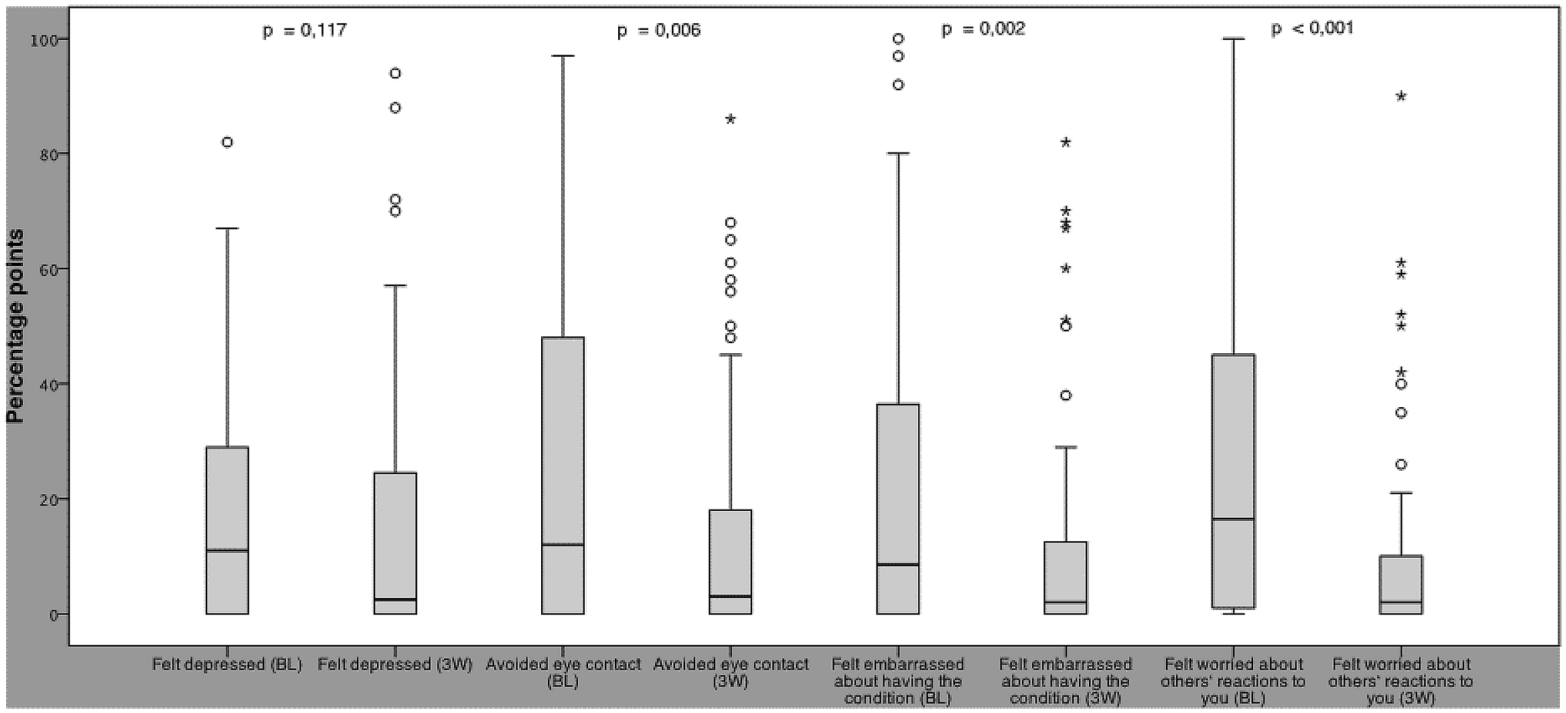
Box plots with whiskers (1.5 × interquartile range (IQR)) of the HFS subjective results on psycological HRQOL on a scale from 0 (no) to 100 (maximum complaints)% points at baseline (BL) and 3 weeks (3 W) after BoNT-A injection. O indicate mild outliers < 3 × IQR; *indicate extreme outliers

The HFS subjective (second part of the HFS score questionnaire) comprises eight questions on HRQOL. With the exception of “had difficulty driving”, all questions of the HFS subjective were answered by all patients. 14 patients stated they did not drive a car and could not answer this question. The results of the HFS subjective in terms of general and physical complaints are shown in Fig. 2. Under BoNT therapy, patients found that their complaints improved in all areas. Significant improvements were observed for “general complaints” (*p* < 0.001; Wilcoxon test), which decreased in median by 26% points from a median of 45 to 19% points, and for “had difficulty reading”, which improved from a median of 35 to 15% points (*p* = 0.001; Wilcoxon test) (Fig. 2). Results of HFS subjective with regard to psychosocial complaints are shown in Fig. 3. Of the four items measuring mental well-being, three showed significant improvements. The most marked absolute improvement was achieved in “felt worried about others’ reactions to you” with a median of 16.5% points, reflecting a relative improvement of 41% (*p* < 0.001; Wilcoxon test). “Felt embarrassed about having the condition” improved significantly by in median 6.5% points from 8.5% points at baseline to 2% points 3 weeks after BoNT-A injection (*p* = 0.02; Wilcoxon test), representing a relative improvement of 75%, “avoid eye contact” improved from 14.5 to 3.0% points after BoNT-A-therapy (*p* = 0.006; Wilcoxon test), while “felt depressed” also improved, however, not significantly.

### Internal consistency of HFS subjective

The reliability analysis to evaluate internal consistency of the HFS subjective resulted in a Cronsbach’s alpha of 0.818. To check if any item of the HFS subjective is inconsistent with the overall result, an item-total correlation test was performed for each question of the HFS subjective. Moreover, for each question it was examined whether its omission would lead to an increase in the Cronbach’s alpha, i.e. to an increase in the internal consistency of HFS subjective. The results are summarised in Table 2 and indicate that the items “had difficulty driving a car” and “had difficulty watching television” exhibit a lower correlation to the overall result than other items. The omission of these two questions from the HFS subjective led to a higher Cronbach’s alpha, while the omission of all other items resulted in a lower Cronbach’s alpha.

**Table 2:** Internal reliability analysis for HFS subjective

	Correlation value^a^	Cronbach’s alpha, if item is deleted
General complaints	0.688	0.777
Had difficulty driving	0.306	0.827
Had difficulty reading	0.627	0.784
Had difficulty watching television / movie	0.199	0.836
Felt depressed	0.515	0.801
Avoided eye contact	0.602	0.788
Felt embarrassed about having the condition	0.701	0.769
Felt worried about others’ reactions to you	0.660	0.778

^a^Item-total correlation test

### Correlation of HFS clinical and HFS subjective

At baseline, the total score for spasms of the eye correlated significantly with “general complaints” (*r* = 0.540; *p* < 0.001), “had difficulty watching TV “ (*r* = 0.269; *p* = 0.034), “felt depressed (*r* = 0.25; *p* = 0.049), “avoided eye contact” (*r* = 0.256; *p* = 0.045) and “felt worried about others’ reactions to you “ (*r* = 0.309; *p* = 0.015). The total score for spasms of the cheek correlated with “general complaints” (*r* = 0.251; *p* = 0.049). The total score for spasms of the eye and cheek correlated with “general complaints” (*r* = 0.397; *p* = 0.01) and “felt depressed” (*r* = 0.256; *p* = 0.045)”. After BoNT-therapy, significant positive correlations were observed between HFS clinical and all items of the HFS subjective (Table 3).

**Table 3:** Correlation between items of HFS subjective and HFS clinical three weeks after injection of BoNT-A

Items of the HFS subjective	Total score eye spasms	Total score cheek spasms	Total sum score spasms (eye and cheek)
General complaints			
Correlation coefficient	0.466	0.575	0.628
** Significance**	** < 0.001**	** < 0.001**	** < 0.001**
Had difficulty driving			
Correlation coefficient	0.357	0.39	0.414
** Significance**	**0.015**	**0.007**	**0.004**
Had difficulty reading			
Correlation coefficient	0.515	0.388	0.542
** Significance**	**< 0.001**	**0.002**	**< 0.001**
Had difficulty watching TV/movie			
Correlation coefficient	0.419	0.426	0.515
** Significance**	**0.001**	**0.001**	**< 0.001**
Felt depressed			
Correlation coefficient	0.311	0.405	0.428
** Significance**	**0.016**	**0.001**	**0.001**
Avoided eye contact			
Correlation coefficient	0.4	0.489	0.533
** Significance**	**0.002**	**< 0.001**	** < 0.001**
Felt embarrassed about having the condition			
Correlation coefficient	0.268	0.450	0.443
** Significance**	**0.038**	**< 0.001**	** < 0.001**
Felt worried about others’ reactions to you			
Correlation coefficient	0.310	0.428	0.443
** Significance**	**0.016**	**0.001**	**< 0.001**

### Reproducibility and stability of the HFS score

The reproducibility and stability of the new HFS score questionnaire were examined in a study extension, which allowed to compare the results obtained by telephone interview with those obtained by an experienced investigator in an examination and personal interview. The 10 patients of this study extension matched well with the other 52 patients in terms of age and gender, as well as to the results of HFS clinical and HFS subjective (at baseline and after 3 weeks of BoNT-A therapy) and can, therefore, be considered representative. Overall, the study extension showed that the results of the HFS score questionnaire obtained in a telephone interview matched well with the assessment of an experienced investigator obtained in an examination and a face-to-face interview 2 days later. The evaluation of clinical parameters using HFS clinical did not reveal any significant differences between the two interview techniques (telephone/personal) (Wilcoxon test).

The results for median, minimum, and maximum collected by telephone 2 days before the BTX therapy, correspond with the results of the personal interview on the day of the BTX therapy.

Moreover, the VAS scales of the HFS subjective completed by the patients showed only small differences between the two interview techniques. There were no significant differences between the interview techniques for the items of HFS subjective for the assessment of physical and mental HRQOL (Wilcoxon test). Nine out of ten patients exhibited differences of ≤ 10% points between the two interview techniques. Only one patient showed differences of > 10% points between telephone and personal interviews in four questions. For “general complaints”, there was a median difference of 11.5% points between the two interview techniques (*p* = 0.013; Wilcoxon test).

### Comparison of HFS subjective and SF-12 in patients and control

Using HFS subjective, a significantly worse quality of life was measured in HFS patients compared to the healthy age- and gender-adjusted control group (*p* < 0.001; Mann–Whitney *U* test). While healthy volunteers achieved a mean score of 8.3% points on the physical composite score (PCS) of the HFS subjective, this was 29.5% points for HFS patients (*p* < 0.001, *t* test). With regard to the mental composite score (MCS) of the HFS subjective, a mean of 2.5% points was reached in the healthy control group, while this was 23.3% points in HFS patients (*p* < 0.001, *t* test).

The SF-12 questionnaire was answered by 57 HFS patients. These results were compared with SF-12 data sorted by age group from a German standard population as a control group (Bullinger und Kirchberger 1998). Regarding the PCS of SF-12, no significant differences were found between HFS patients and the standard population. Regarding MCS of SF-12, HFS patients of all age groups displayed worse MCS values compared to the standard population, however, statistically significant differences were observed only for the age group from 61 to 70 years (*p* = 0.038) and for the total group (*p* = 0.018).

## Discussion

In the present study the newly developed, specific and sensitive HFS score questionnaire was validated and successfully used to assess the therapeutic effect of BoNT-A therapy in HFS patients. The first part (HFS clinical) assesses clinical parameters, while the second part (HFS subjective) focuses on functional and psychosocial HRQOL aspects of the disease. Considerations that led to its development have already been described in detail together with a table with rating scales used for the evaluation of HFS, some of which were not developed specifically for HFS (Wabbels and Roggenkämper 2012). Table 4 compares the here presented HFS score with the key parameters of newer rating scales developed specifically for the HFS and with the SF-12 to enable their rapid and objective comparative assessment. The HFS score is the first questionnaire covering all relevant aspects for a complete assessment of HFS—clinical as well as subjective HRQOL parameters—in one single and easy to perform questionnaire. In addition, as our results show, the questionnaire can also be used by telephone and allows detection of even small changes due to the VAS used.

**Table 4:** Rating scales designed for hemifacial spasm (HFS) and comparison with the SF-12/36

	Validation	Clinical assessment	HRQOL assessment	Special features
HFS score This publication	62 HFS patients vs. 81 healthy subjects Discrimination disease/control Internal consistency Correlation with SF-12	Eye and cheek spasms Frequency and severity Each item by 5-point rating scale (0–4) Max Score: 16	One item on general complaints 7 HFS specific HRQoL items (HFS-7) Subjective assessment by VAS	Quick clinical and HRQoL assessment specific to HFS Applicable by telephone Allows detection of small changes (VAS)
HFS-7 Tan EK et al. (2005)	85 HFS patients vs. 93 healthy subjects Discrimination disease /control Internal consistency Correlation with SF-36	No clinical assessment	7 HRQoL items covering problems specific to HFS subjective assessment by 5-point rating scale	Quick and subjective assessment of HFS specific HRQoL
HSGS Tambasco N et al. (2019)	36 HFS patients Intra- and inter-rater reliability based on video recordings No correlation to HSF-7 observed	Upper/lower face muscles or both Frequency and severity Different scores for each single item Max Score: 9	No HRQoL assessment	Quick and objective clinical assessment
SF-12/SF-36 Bullinger et al. (1998)	Well-established questionnaires for generic HRQOL	No clinical assessment	Subjective and generic HRQoL assessment No specific items on HFS	No specific items for HFS

*HRQoL* health related quality of life, *VAS* visual analogue scale

The large patient population recorded in this study reflects a typical population of HFS patients and can be considered appropriate for the validation of this new questionnaire. Twice as many female as male patients were included, which is in line with the results of other studies on HFS (Auger und Whisnant, 1990). The average age was slightly higher than in many other studies, at about 70 years, which is probably also due to the slightly longer therapy duration of a mean of 8.8 years compared to other studies (Chen et al., 1996; Poungvarin et al., 1995; Rieder et al., 2007; Tunç et al., 2008; Wang und Jankovic, 1998; Yoshimura et al., 1992).

### Clinical parameters assessed by HFS clinical

The aim of symptomatic BoNT-A therapy is to reduce hemifacial spasms of eye and cheek in HFS patients. To measure the effect of BoNT-A, therefore, a clinical instrument is required that enables the examiner to sensitively measure the reduction of spasms in the eye and cheek. Many of the instruments that have been used to date to measure spasm intensity in HFS patients consist of a 5-point scale with a basic description (Chen et al., 1996; Park et al., 1993; Tan et al., 2004; Tunç et al., 2008). While they are easy to apply, they often do not capture anatomical manifestation, frequency and severity of spasms and therefore do not provide a precise and specific assessment, so several independent investigators may come to different conclusions about the severity of the condition. The JRS measures both frequency and severity of eye spasms using a 5-point scale and is established as a recognised clinical tool for measuring eye spasms in essential blepharospasm (Wabbels et al. 2011). Due to its successful application in essential blepharospasm, it also appears to be suitable for the assessment of HFS spasms. In our clinical experience, spasms of the eye can often be treated more effectively than those of the cheek, which may then remain as a source of subjective HRQOL impairment for patients. Therefore, not including cheek spasms in the physician’s clinical assessment could lead to unexplained discrepancies between the patient’s and physician’s assessment of improvement after treatment. For this reason, the HFS clinical is based on the JRS for evaluating the frequency and severity of eye spasms and has been extended to also evaluate the frequency and severity of cheek spasms (see Supplementary file). Using HFS clinical, highly significant improvements were observed in both eye and cheek spasms after BoNT-A therapy (Fig. 1), indicating that HFS clinical is an effective assessment tool for clinical measurement of spasms in HFS patients. While the HFS clinical allows to reliably measure the clinical success of BoNT-A therapy compared to baseline, it remains unclear whether this categorical scale is also sufficient for addressing other research purposes. The 5-point scale may not be able to distinguish between small differences in severity and frequency of spasms. However, these small differences might be relevant to the patient’s subjective assessment or to the distinction between various BoNT formulations. In addition, for direct comparability of the results of both parts of this questionnaire (HFS clinical and HFS subjective), values of the 0–16 scale of the HFS clinical could be converted into percentages (resulting in intervals of 0, 6, 12, 19, 25% …).

### HRQOL parameters assessed by HFS subjective

HFS is a chronic disease that regularly leads to social isolation in addition to physical limitations. Measuring the clinical severity of the spasms as the sole criterion for therapy success is therefore not sufficient; rather, the subjective impairment of the patient must also be taken into account.

Up to now, global rating tools have often been used to assess HRQOL aspects of HFS, however, these are not disease-specific and often utilize basic ordinal scales for subjective self-assessment. The newly developed HFS subjective validated here is specifically designed to capture HRQOL aspects related to HFS. It consists of an introductory question on general complaints as well as three questions on functional and four questions on psychosocial HRQOL, which are based on the HFS-7 specially developed for HFS (Tan et al. 2005). Unlike the HFS-7 by Tan et al. (2005), assessment of questions in HFS subjective is based on a visual analogue scale (VAS). This newly developed HFS subjective allowed to observe improvements in all HRQOL areas 3 weeks after BoNT-A injection, although the quality of life of our HFS patients was only moderately impaired at baseline. Even significant improvements were achieved in two functional HRQOL items (“general complaints”; “reading”) and in three of four psychosocial HRQOL parameters (“felt worried about others’ reactions to you”; “felt embarrassed about having the condition”; “avoid eye contact”). VAS are recognised to enable a more sensitive recording of complaints and are established as standard instrument in pain assessment (Bjur et al. 2001). Especially in our HFS population with an initially only moderately limited HRQOL, the ability of VAS to detect even small changes was particularly valuable to reliably assess therapy success: The results of four questions on psychological complaints both before and after BoNT-A therapy ranged between 0 and 20% points in the median. On a 5-point scale, one step corresponds to a change of 20% points on the VAS, so no difference would have been detected in these cases. However, the VAS scale used here was able to identify even these small changes. If a 5-point scale is used, the choice of answers may not be sufficient to accurately reflect the patient’s assessment. Patients may then be inclined to choose the most appropriate answer, but this may still cause some bias in the results.

Moreover, in this study we could demonstrate that the HFS subjective has discriminating power to distinguish HFS patients from healthy subjects. Results were highly significant (*p* < 0.001) for both the physical composite score (PCS) and the mental composite score (MCS), indicating that HFS subjective is very effective to distinguish between healthy and HFS patients. Overall, our results demonstrate that HFS subjective can be used to measure the quality of life of HFS patients with a high degree of accuracy, whereas generic instruments such as SF-12 are, as expected, not sufficiently sensitive to measure the HRQOL of HFS patients and can only measure partial aspects at best.

### Internal consistency of the new HFS questionnaire, correlation of HFS clinical and HFS subjective

In general, a Cronbach’s α of at least 0.7 is considered sufficient to assume an internally consistent scale. Therefore, the HFS questionnaire with a Cronbach’s α of 0.818 showed good internal consistency, indicating that all questions can be meaningfully combined in one single questionnaire. Although the internal consistency of the HFS would increase if the items “difficulty watching television” and “difficulty driving” were omitted, these two questions should nevertheless remain in the HFS questionnaire, as it is important to consider both reliability and relevance when designing a questionnaire. During HFS validation, we first determined which activities are of particular relevance for HFS patients. In accordance with the results of (Tan et al. 2005), we found that for most HFS patients reading, watching television and driving a car are the most important activities. They should therefore be addressed in an HFS questionnaire. Furthermore, in agreement with other studies (Tan 2005; Cheng 2017; Lee 2012), our study also found a positive correlation between clinical severity and reduced HRQOL in HFS patients. While the correlation with HFS clinical was not present for all HFS subjective questions at the beginning of the study, they all correlated significantly with HFS clinical after BoNT-A therapy. Overall, the significant correlations between the HFS clinical and HFS subjective demonstrate a good agreement between the clinical severity and subjective perception of HFS patients after BoNT-A therapy and provide a good indication of the benefit of BoNT-A therapy in HFS.

### Reproducibility and stability of the new HFS questionnaire

In the study extension, the results obtained in telephone interviews were confirmed by the assessment of experienced investigators conducted 2 days later in our clinic, indicating that patients were able to reliably assess frequency and severity of their cheek and eye spasms themselves using the HFS clinical with telephone guidance. Also, the telephone interview on HRQOL using HFS subjective provided comparable results to the personal interview. This suggests that the new HFS score questionnaire can be applied in both telephone and personal interviews and provides comparable and reproducible results. Even though a significant difference was found between the two interview techniques with regard to “general complaints”, this was nevertheless relatively small (in median 11.5% points) and would not be detected with a 5-point scale. This option to conduct telephone interviews is a key advantage of this questionnaire—both for everyday clinical practice and for clinical studies. Due to their age, many HFS patients often cannot be expected to present again on site for an assessment of the BoNT effect. A telephone survey enables uncomplicated monitoring of therapy success, especially in rural areas. In addition, it also facilitates a longer follow-up in clinical studies on BoNT-A therapy or microvascular decompression surgery, as patients no longer need to come to the study centre, but the success of the therapy can also be evaluated by telephone.

## Limitations

The results presented here were obtained in a German population. It remains unclear whether this instrument also covers the most important HRQOL aspects of other HFS populations, also with regard to cultural differences. Second, the therapy duration was relatively long in our study population, with an average of 8.8 years. Habituation factors after prolonged therapy duration may result in better HRQOL to baseline than after a shorter therapy duration (Tan 2005). This effect should be investigated in trials with a larger group of therapy naive patients. Third, the adverse events in our study were analysed retrospectively before the next injection. Although these were small, they may be a burden to individual patients and may affect HRQOL. Therefore, the questionnaire should be supplemented with questions about the adverse effect profile for future studies.

## Conclusion and outlook

The newly developed HFS score questionnaire demonstrated a highly significant reduction of spasms of the eye and cheek in our HFS population under BoNT therapy and an improvement in relevant aspects of HRQOL. In addition, the new HFS score showed good internal consistency as well as high reproducibility and stability. Even when conducted by telephone, it provided reliable results comparable to those obtained by face-to-face interviews. Therefore, the new HFS score, including clinical and HRQOL parameters, seems an appropriate tool for use in clinical practice to monitor BoNT therapy and customize it to the needs of the individual HFS patient. It can also be used in clinical trials to collect long-term data on BoNT therapy or microvascular decompression surgery and to evaluate small differences in the effects of different therapeutics.

## Supplementary Information

Below is the link to the electronic supplementary material.

Supplementary file1 (PDF 116 KB)

## Data Availability

BW and AY had full access to all the data in the study and take responsibility for the integrity of the data and the accuracy of the data analysis. BW and AY conducted and are responsible for the data analysis. Original data will be shared from the corresponding author on reasonable request.
