# Supplementary material for: Validation of a new hemifacial spasm grading questionnaire (HFS score) assessing clinical and quality of life parameters
Source: J Neural Transm (Vienna). 2021 May 8;128(6):793–802. doi: 10.1007/s00702-021-02343-x (PMC8205881; doi:10.1007/s00702-021-02343-x)
Supplement: Supplementary file 1 — Supplementary file1 (PDF 116 KB) [file 702_2021_2343_MOESM1_ESM.pdf]

Validation of a new hemifacial spasm grading questionnaire (HFS score) assessing clinical and quality of life parameters

Bettina Wabbels[1], Ali Yaqubi,[1,2]

1 Department of Ophthalmology, University of Bonn, Ernst-Abbe-Str. 2, 53127 Bonn, Germany

2 Ophthalmological Center Lippstadt, Wiedenbrücker Str. 31, 59555 Lippstadt, Germany

Corresponding author:

Prof. Dr. med. Bettina Wabbels, FEBO

ORCID ID: 0000-0002-7767-114X

Tel.: +49 (0)228-287 15612

Fax: +49 (0)228-287 14692

Email: bettina.wabbels@ukb.uni-bonn.de

Hemifacial spasm grading questionnaire: HFS score

1. **HFS clinical**
2. **HFS subjective**

(1 a) HFS clinical: Eye involvement

**Hemifacial Spasm Severity**	**Frequency**
0 = None<br>1 = Minimal, increased blinking present <u>only</u> with external stimuli (e.g., bright light, wind, reading, driving, etc.)<br>2 = Mild, but spontaneous eyelid fluttering (without actual spasm), definitely noticeable, possibly embarrassing, but not functionally disabling)<br>3 = Moderate, very noticeable spasm of eyelids only, mildly incapacitating<br>4 = Severe, incapacitating spasm of eyelids and possibly other facial muscles	0 = None<br>1 = Slightly increased frequency of blinking<br>2 = Eyelid fluttering lasting less than 1 second [illegible] duration<br>3 = Eyelid spasm lasting more than 1 second, eyes open more than 50% of the waking ti[illegible]<br>4 = The involved eye is functionally “blind” due[illegible] persistent eye closure more than 50% of th[illegible] waking time

(1 b) HFS clinical: Cheek involvement

**Severity**	**Frequency**
0 = None<br>1 = Mild, barely noticeable spasm, only recognised by the patient<br>2 = Mild, but noticeable spasm<br>3 = Moderate noticeable spasm including the corners of the mouth<br>4 = Severe spasm with involvement of the whole cheek	0 = None<br>1 = Slightly increased frequency of cramps<br>2 = Cramps lasting less than 1 second in durat[illegible]<br>3 = Cramps more than 1 second<br>4 = Cramps more than 50% of the waking time

(2) HFS subjective: Health-related quality of live

(1) Global rating

Free of complaints suffering extremely

0% I____I 100%

(2) Had difficulty driving

0% I____I 100%

(3) Had difficulty reading

0% I____I 100%

(4) Had difficulty watching television / movie

0% I____I 100%

(5) Felt depressed

0% I____I 100%

(6) Avoided eye contact

0% I____I 100%

(7) Felt embarrassed about having the condition

0% I____I 100%

(8) Felt worried about others' reactions to you

0% I____I 100%
